# Necrotizing enterocolitis in a neonate with severe congenital pulmonary valve stenosis complicated by a postoperative right atrial thrombus: a case report

**DOI:** 10.3389/fped.2025.1594899

**Published:** 2025-12-03

**Authors:** Wenjing Zhang, Li Zhang, Haiting Liu

**Affiliations:** 1 Department of Pediatrics, West China Second University Hospital, Sichuan University, Chengdu, Sichuan, China; 2 Key Laboratory of Birth Defects and Related Diseases of Women and Children, Sichuan University, Ministry of Education, Chengdu, China

**Keywords:** pulmonary valve stenosis, necrotizing enterocolitis, metagenomic next-generation sequencing, anticoagulation, case report

## Abstract

Congenital heart disease may increase the incidence of necrotizing enterocolitis, especially in low-birth-weight infants. We report a case of a newborn with pulmonary valve stenosis who developed neonatal necrotizing enterocolitis IIIB. The infant underwent initial cardiac surgery followed by a laparotomy. After cardiac surgery, a right atrial thrombus was found by cardiac ultrasound. *Enterococcus faecium* was identified using metagenomic next-generation sequencing of ascitic fluid. The infant received targeted antibiotic therapy and anticoagulant treatment and was then discharged.

## 1 Introduction

Neonatal necrotizing enterocolitis (NEC) is one of the most severe acquired intestinal diseases in neonates and is a common complication in preterm infants, especially in very-low and extremely-low birth-weight infants, occurring in approximately 10% at term (1, 2). Among these, NEC induced by congenital heart disease (CHD) accounts for approximately 20% of cases of term infants with NEC (3, 4). We present the case of a full-term infant with severe pulmonary valve stenosis whose intensive care course was complicated by the development of NEC.

## 2 Case presentation

**Figure 1:**
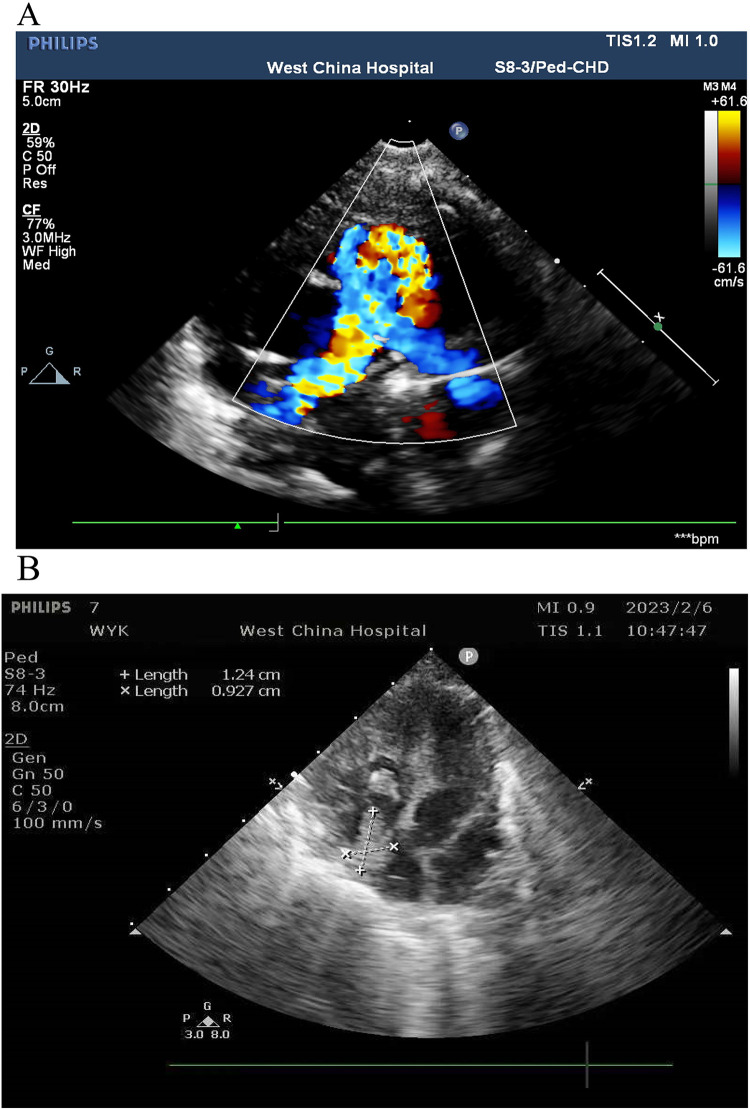
**(A)** Preoperative cardiac ultrasound. **(B)** Postoperative cardiac ultrasound.

**Figure 2:**
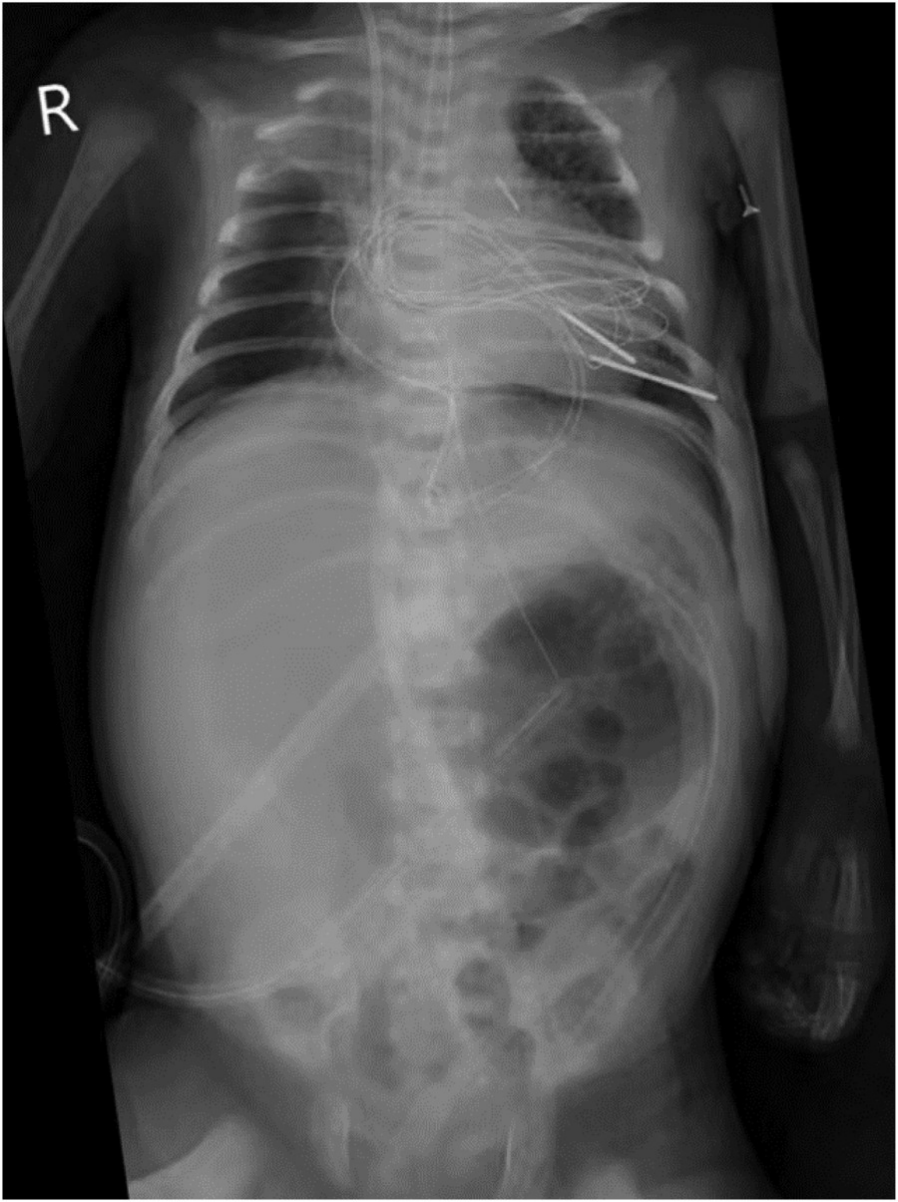
Abdominal X-ray.

An 11-day-old female neonate was diagnosed with severe pulmonary valve stenosis during the fetal period. She was born at 40 1/7 weeks' gestation with a birth weight of 3,450 g and was admitted to another hospital upon birth for prostaglandin E1 (alprostadil) treatment to maintain patency of the ductus arteriosus (PDA). After 1–2 days, she developed dyspnea, abdominal distension, and oliguria, and was then transferred to a different hospital. The preoperative cardiac ultrasound revealed severe pulmonary valve stenosis (Figure 1A). Subsequently, due to the large PDA, surgical repair of the pulmonary valve stenosis, arterial catheter ligation, and tricuspid valvuloplasty were performed under cardiopulmonary bypass. During the surgery, the partial valve was resected, and the pulmonary artery incision was closed with an autologous pericardial patch to augment the pulmonary artery sinus. Postoperative cardiac ultrasonography revealed an approximately 12 mm × 9 mm right atrial thrombus (Figure 1B), for which low-molecular-weight heparin was administered for anticoagulation. “The preoperative cardiac ultrasound was normal and is shown in Figure 1A”. The cardiac ultrasound shows severe pulmonary valve was stenosis rather than normal. The infant received two central catheters that were placed in the right femoral vein and left femoral artery, respectively. However, 6 days after cardiac surgery, her abdominal symptoms worsened. Abdominal paracentesis revealed ascitic fluid with bile, and an abdominal X-ray suggested a gastrointestinal perforation (Figure 2). An abdominal drainage tube was inserted. After 48 h of worsening symptoms, she was transferred to our hospital.

**Figure 3:**
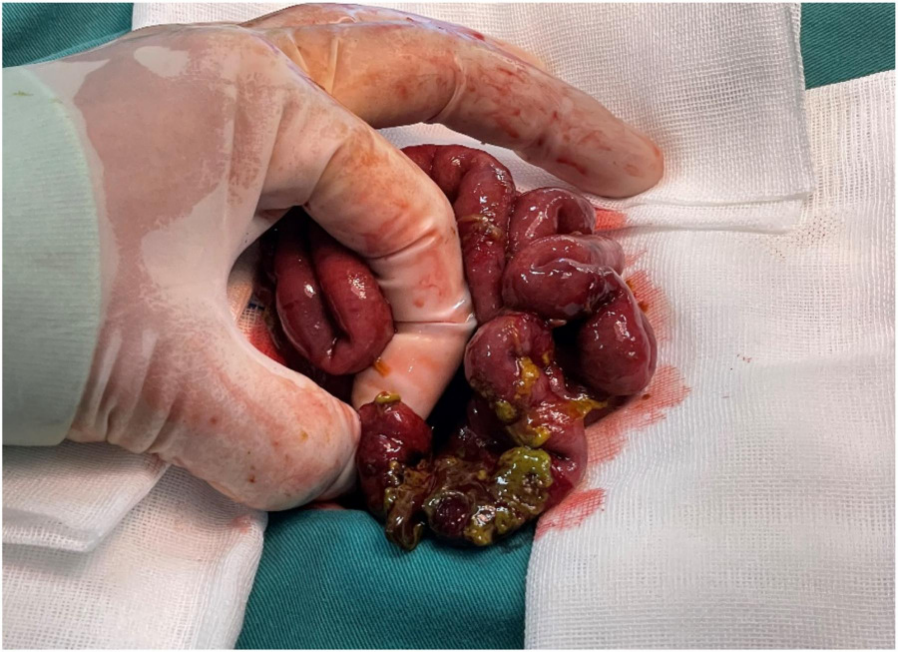
Intraoperative small bowel perforation.

Medications were administered, and the infant was intubated and mechanically ventilated. The medications included vancomycin, meropenem, and low-molecular-weight heparin. Abdominal distention persisted after the abdominal drainage. Thus, 3 days after admission, she underwent an exploratory laparotomy, intestinal resection, and ileostomy. Ink green ascites, which contained feces and purulent debris, were found internally. There was a necrotic perforation of the ileum approximately 5 cm from the ileocecal site (Figure 3), and a 5-cm fistula was created after resection of the necrotic ileum. She was diagnosed with necrotizing enterocolitis IIIB.

**Figure 4:**
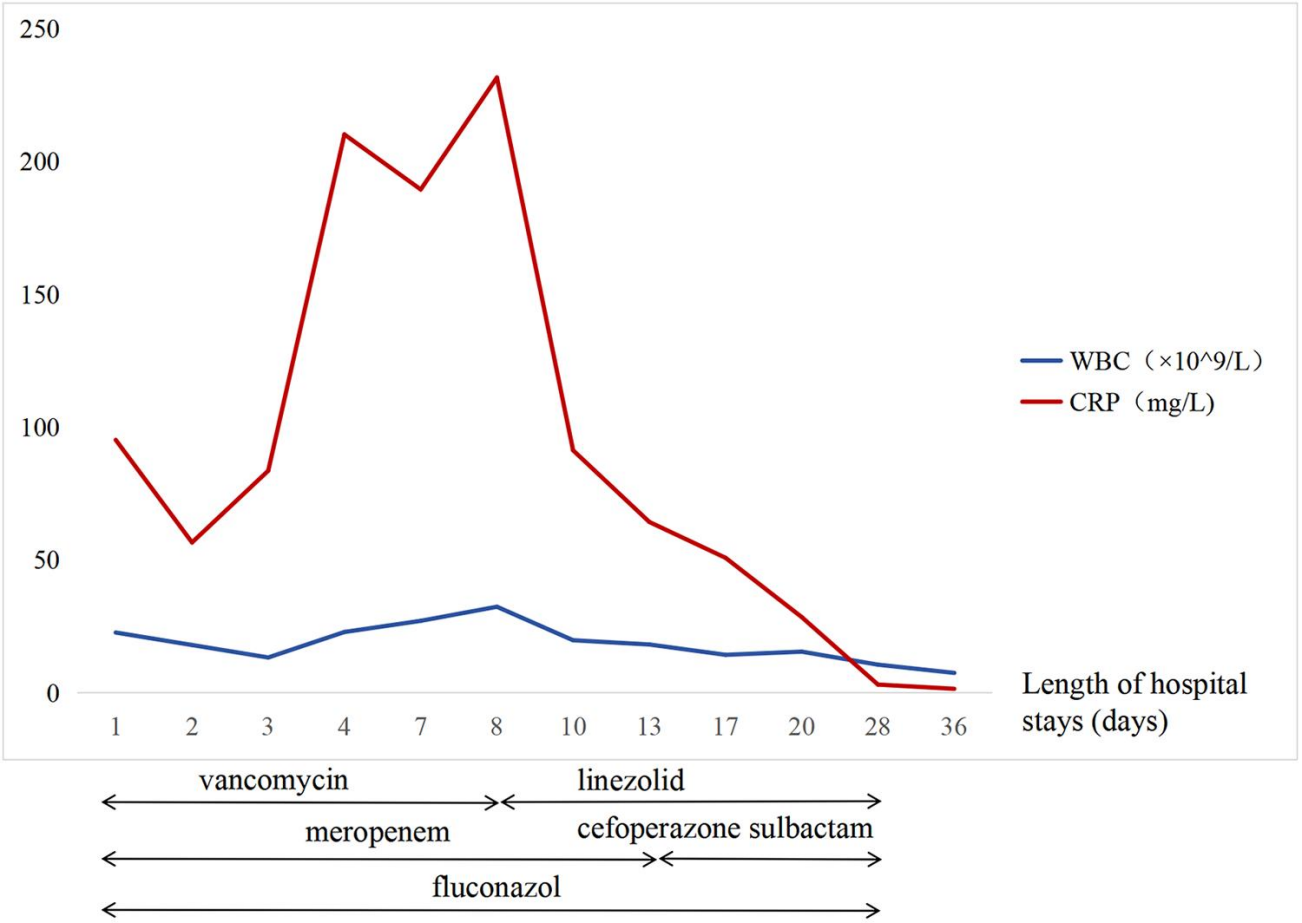
White blood cell and CRP trends.

*Enterococcus faecium* was detected in the ascitic fluid using metagenomic next-generation sequencing (mNGS). However, the blood and ascitic fluid cultures were negative. She developed a fever, and her C-reactive protein (CRP) level was markedly elevated to 231 mg/L on the 10th day of hospitalization. The possibility of vancomycin-resistant *E. faecium* was considered, and linezolid (10 mg/kg/time, Q8 h) was administered. Her temperature returned to normal, and the CRP level gradually decreased to a normal level (Figure 4). Antibiotics were discontinued after 3 weeks.

The patient was monitored for disseminated intravascular coagulation, thrombus formation, and anti-Xa activity. The patient exhibited marked elevations in the thrombin-antithrombin complex (TAT) (9 ng/mL, normal range <4.0 ng/mL) and α2-plasmininhibitor-plasmin complex (PIC) (2.1 µg/mL, normal range <0.8 µg/mL). Fresh frozen plasma was repeatedly infused. Low-molecular-weight heparin was administered with close monitoring of coagulation function and adjustment of the dose according to anti-Xa activity. The initial recommended dose of low-molecular-weight heparin for term infants is 1.7 mg/kg/time, ih, q12h (5). Anti-Xa activity was measured 4–6 h after the second dose was administered, with a target range of 0.5–1 units/mL. Repeat heart ultrasonography showed no right atrial thrombus after 35 days of anticoagulant therapy.

As for nutritional management, the infant was continuously maintained nil per os from the initial onset of abdominal distension after birth until the 7th day after the laparotomy, and received total parenteral nutrition for 23 days. The total parenteral nutrition incorporated a compounded intravenous lipid preparation comprising soybean oil, medium-chain triglycerides, olive oil, and fish oil (SMOF) for the maintenance of vital functions and growth. SMOF was used for enteral nutrition to avoid the development of intestinal failure-related liver disease because the direct bilirubin level of the neonate was <2 mg/dL. The decision to resume enteral feeding after withholding feeds was mainly based on an assessment of the infant's clinical status, which included improved respiratory support, hemodynamic stability, absence of significant abdominal distention or vomiting, and radiography showing no notable bowel dilation or pneumatosis. The early initiation of trophic feeding could prevent gastrointestinal atrophy, facilitate intestinal maturation, and help preserve gut barrier function, thereby reducing the risk of allergic enterocolitis. An extensively hydrolyzed formula was administered due to the lack of breast milk, first via a nasogastric tube and then transitioning to oral intake. The patient underwent fistula surgery 2 months later. After 2 years of follow-up, the patient exhibited normal growth and development.

## 3 Discussion

### 3.1 The many risk factors for CHD-NEC

NEC can occur pre- or postoperatively in infants with CHD, and the presence of congestive heart failure, systemic to pulmonary arterial shunts, or cyanotic CHD-NEC is associated with higher morbidity and mortality. The presence of CHD in neonates with a birth weight >2,500 g is an independent risk factor for NEC (6–8). The potential pathogenesis and etiology of CHD-NEC include mesenteric hypoperfusion (sustained/intermittent), hypoxia-induced inflammation, surgical stress (cardiopulmonary bypass/hypothermia), ductal-dependent congenital heart lesions, and prolonged use/high-dose alprostadil treatment (9). This type of NEC predominantly occurs in full-term and near-term infants, with an onset typically earlier than classical NEC (10). The necrotic lesions of CHD-NEC have a predilection for the colon and distal ileum, as these areas are vulnerable to impaired perfusion within the watershed zones of the superior and inferior mesenteric arteries (11). The reported associated risk factors for CHD-NEC are prematurity, low birth weight, low cardiac output, atrioventricular septal defect, red blood cell transfusion, prostaglandin use, and undergoing cardiopulmonary bypass (4). Prostaglandin E1 is effective in opening a PDA (12). As infants mature after delivery and pulmonary vascular resistance drops over time, maintaining a PDA with prostaglandins increases pulmonary blood flow and decreases systemic blood delivery, leading to pulmonary edema and increasing the risk of decreased perfusion to the intestines, which may be a risk factor for NEC. Although prostaglandin E1 has been considered a risk factor for NEC, the underlying cardiac lesion is more likely the primary contributor in this case. Moreover, the oliguria in this case is likely multifactorial, including impaired venous return due to congenital heart disease and infection-related factors.

Hemodynamic monitoring, near-infrared spectroscopy, and a standardized physical examination score could help identify and diagnose NEC earlier and be used to evaluate surgical indications in these patients (13). The score is calculated based on the following physical examination: abdominal girth ≥1 cm, abdominal erythema, abdominal discoloration, tenderness, no bowel sounds for 1 min, capillary refill time (CRT) ≥2 s, and induration. CRT of 2–3 s is assigned one point, while CRT exceeding 3 s is assigned two points; all the other physical examination findings are assigned one point each. A total score of ≥3 was highly sensitive and specific for determining the composite outcome of the need for surgery or death from NEC prior to surgery (14).

### 3.2 mNGS application and neonatal VRE

Previous studies by us and other authors have found that compared to traditional culture, mNGS demonstrates higher sensitivity and accuracy in pathogen detection (15–19). Thus, in our NICU, we perform mNGS using bodily fluids, such as bronchoalveolar lavage fluid or ascitic fluid, to complement blood samples, so as to improve the probability of a positive diagnosis and optimize therapy for critically ill newborns (16). We suggest the use of mNGS in different specimens, which could expedite diagnosis and treatment in critical patients. In this case, *E. faecium* was only detected by ascitic fluid mNGS. However, due to the high cost of mNGS, it is not suitable for all patients. In addition, it does not provide antimicrobial susceptibility testing results, so it still cannot replace traditional cultures.

Vancomycin is frequently employed to treat *E. faecium*; however, the infant was repeatedly febrile with a progressive increase in CRP levels during the administration of vancomycin. The known risk factors for VRE colonization include long hospital stays, the need for intensive care, exposure to invasive procedures, and receipt of broad-spectrum antibiotics (20). Accordingly, the patient was at a high risk of VRE colonization and infection. The patient consequently received intravenous linezolid, leading to her fever subsiding and her inflammatory markers improving.

### 3.3 Monitoring anticoagulation treatment for a right atrial thrombus

The patient developed a right atrial thrombus after undergoing cardiac surgery, which may have been associated with disseminated intravascular coagulation due to central venous catheterization (CVC) puncture and endothelial injury caused by sepsis. For children with right atrial thrombi associated with a central venous access device, eradication of the device is recommended along with anticoagulant therapy. Thrombophilia testing is not routinely performed in patients with a first episode of CVC-related venous thromboembolism. The parents of the patient had no history of embolism (21). The assays of four thrombotic biomarkers include TAT, thrombomodulin (TM), PIC, and tissue plasminogen activator-plasminogen activator inhibitor complex (t-PAIC). TAT reflects the amount of thrombin generated or the extent of coagulation activation, and its elevation suggests a hypercoagulable state with a high risk of thrombosis. PIC reflects the amount of plasmin generated or the degree of fibrinolytic system activation and an elevation suggests hyperfibrinolysis. TM is widely utilized as a biomarker, reflecting endothelial cell integrity and function, while t-PAIC serves as a specific molecular marker indicative of fibrinolytic activity, endothelial cell injury, and multiple organ failure. Compared to conventional coagulation tests, these assays offer significantly higher sensitivity for non-dominant disseminated intravascular coagulation (DIC) and can assess coagulation and fibrinolytic systems, as well as the condition of endothelial injury. The use of these assays could result in earlier anticoagulation and improved outcomes (22–24).

The timing of the testing is determined based on the clinical condition of the infant on 2–3 occasions and both these assays and usual coagulation laboratory tests can be run from the same blood sample, thereby avoiding excessive blood loss and hospital-acquired anemia. Our previous study suggested that these assays appeared to predict the early phase of DIC, guide anticoagulant therapy effectively, and reduce the incidence and mortality of venous thrombosis (16).

## 4 Conclusions

CHD, which predisposes patients to NEC, has an earlier age of onset than preterm birth and is associated with high mortality. Prolonged high-dose alprostadil treatment should be administered cautiously in neonates with ductal-dependent CHD; otherwise, closely monitoring gastrointestinal signs and symptoms is required. For critically ill neonates, it is recommended to utilize mNGS for pathogen identification and to facilitate clinical diagnosis and treatment. Moreover, monitoring coagulation function via four thrombotic biomarkers, namely, TAT, PIC, TM, and tPAIC, is recommended in critically ill neonates.

## Data Availability

The original contributions presented in the study are included in the article/Supplementary Material, further inquiries can be directed to the corresponding authors.
