# Correction: Necrotizing enterocolitis in a neonate with severe congenital pulmonary valve stenosis complicated by a postoperative right atrial thrombus: a case report

**DOI:** 10.3389/fped.2025.1760028

**Published:** 2026-01-02

**Authors:** Wenjing Zhang, Li Zhang, Haiting Liu

**Affiliations:** 1 Department of Pediatrics, West China Second University Hospital, Sichuan University, Chengdu, Sichuan, China; 2 Key Laboratory of Birth Defects and Related Diseases of Women and Children, Sichuan University, Ministry of Education, Chengdu, China

**Keywords:** pulmonary valve stenosis, necrotizing enterocolitis, metagenomic next-generation sequencing, anticoagulation, case report

**Text correction**

Adding/removing text

In Case presentation section, Page 2, line 121–122 “The preoperative cardiac ultrasound was normal and is shown in Figure 1A”. The cardiac ultrasound shows severe pulmonary valve was stenosis rather than normal.

A correction has been made to the section Case presentation section, Page 1, line 111–112.

“After 1–2 days, she developed dyspnea, abdominal distension, and oliguria, and was then transferred to a different hospital. The preoperative cardiac ultrasound revealed severe pulmonary valve stenosis (Figure 1A). Subsequently, due to…”

The original version of this article has been updated.

